# Whitefly Control Strategies against Tomato Leaf Curl New Delhi Virus in Greenhouse Zucchini

**DOI:** 10.3390/ijerph16152673

**Published:** 2019-07-26

**Authors:** Estefanía Rodríguez, Mª Mar Téllez, Dirk Janssen

**Affiliations:** IFAPA, La Mojonera Centre, 04745 Almería, Spain

**Keywords:** augmentative biological control, *Cucurbita pepo*, parasitoids, predators, protected horticulture, begomovirus

## Abstract

(1) *Background:* Tomato leaf curl New Delhi virus (ToLCNDV), transmitted by tobacco whitefly (*Bemisia tabaci* Gennadius) (Hemiptera: Aleyrodidae), is of major concern in the cultivation of zucchini. The threat of this virus motivates reliance on chemical vector control but European consumers’ demands for vegetables grown free of pesticides provides an important incentive for alternative pest management; (2) *Methods:* Different whitefly management strategies and ToLCNDV incidences were surveyed in commercial zucchini greenhouses in south-east Spain. In an experimental greenhouse, three different whitefly control strategies, biological, chemical, and integrated (IPM), were evaluated in a replicated trial to determine the most effective strategy for virus suppression (3) *Results:* Whitefly was present in all commercial zucchini crops surveyed, whereas fewer crops had *Amblyseius swirskii* or other natural enemies. During three consecutive years, pest management was increasingly based on chemical treatments. Yet, ToLCNDV was widespread in zucchini greenhouses. Experimental results showed that the order of best strategy for virus suppressing was integrated management (73%) > biological control (58%) > chemical control (44%); and (4) *Conclusions:* IPM was the best strategy for virus suppression. The results can assist in the design of appropriate control strategies for chemical pesticide reduction and decision-making in pest management.

## 1. Introduction

The largest horticultural greenhouse producing area in Europe is located in south-east (SE) Spain (31,000 ha). The whitefly, *Bemisia tabaci* (Gennadius) (Hemiptera: Aleyrodidae), is one of the most serious pests found in protected horticulture because of its broad host range, high reproductive rate, and short life cycle [1]. The most frequent damage they cause is due to the hundreds of plant viruses (> 350 species) they are able to transmit, which include members of the genera *Begomovirus* (*Geminiviridae*), *Crinivirus* (*Closteroviridae*), *Ipomovirus* (*Potyviridae*), *Carlavirus* (*Betaflexiviridae*), and *Torradovirus* (*Secoviridae*) [2]. Most economically important viruses are begomoviruses, which have increased their distribution and importance worldwide [3], and are responsible for significant yield losses in Spanish horticulture [4,5].

Traditionally, chemical control has been the dominant strategy in *B. tabaci* management. Consequently, the pest has developed resistance and cross-resistance to a wide range of insecticides in the field. Mediterranean species (MED, also commonly known as biotype Q) is the most predominant and devastating biotype across horticultural crops in SE Spain [5,6]. Moreover, biotype Q shows greater resistance to insecticides compared with the widespread biotype B [7,8]. This likely explains the displacement of biotype B by biotype Q in many Mediterranean countries [9,10]. Adequate climatic as well as agronomic conditions in SE Spain, e.g., year-round greenhouse crop production, favor the presence of high populations of *B. tabaci* and, consequently, the introduction of new begomoviruses [5]. In fact, Spanish horticulture greenhouses have been recently invaded by the bipartite begomovirus, tomato leaf curl New Delhi virus (ToLCNDV), which infects solanaceous and cucurbitaceous crops, but which is especially aggressive in zucchini crops. Since its introduction in 2013, it has caused considerable economic losses in Spain [11,12].

There is a rising demand by European consumers for reduced pesticide use in horticulture due to health reasons and the need to protect the environment [13]. Integrated pest management (IPM) is an interdisciplinary approach for control of agricultural pest populations that is regulated in the European Union Member States (Directive 2009/128/EC). Augmentative biological control is an important component of IPM programs in protected horticulture. The predatory mite *Amblyseius swirskii* Athias-Henriot (Acari: Phytoseiidae) is particularly useful against the whitefly in sweet pepper and cucumber [14,15,16,17,18,19]. Semifield tests showed that zucchini plants harboring *A. swirskii* have lower ToLCNDV incidence with less virus spreading than plants without *A. swirskii* [20]. Moreover, preinstallation of *A. swirskii* on zucchini seedlings supplemented with pollen as food resource allows building up of large populations of predators before the pest arrives [19,20]. Little is known about the current phytosanitary balance and virus-spread in commercial zucchini greenhouses from SE Spain, or which of the whitefly reducing strategies (chemical, biological or IPM) is optimal for virus suppression. In order to avoid pesticide overuse, the first objective of this study was to investigate the recent evolution of *B. tabaci* in commercial zucchini crops under conventional (chemical) and integrated management, and the most recent incidence of ToLCNDV. The second objective was to compare the effectiveness of a biological (particularly, the preinstallation of the predatory mite *A. swirskii*), chemical (conventional management), and integrated strategy in an experimental greenhouse to determine the most successful measure for ToLCNDV suppression in zucchini.

## 2. Materials and Methods

### 2.1. Commercial Zucchini Crop Monitoring

**Figure 1:**
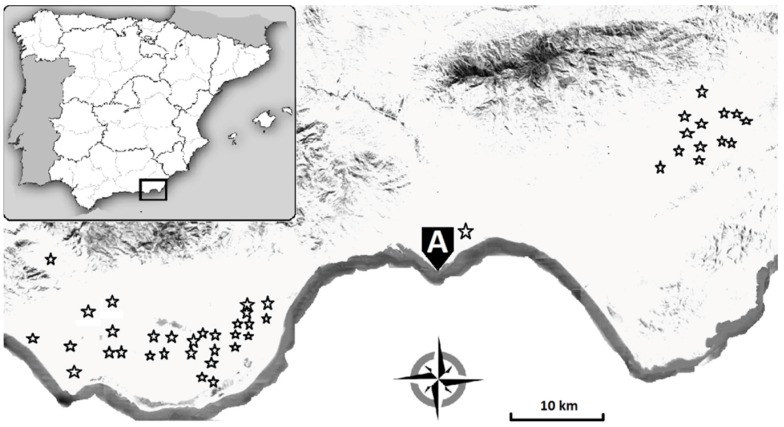
Location of 44 greenhouses surveyed for monitoring whitefly-control strategies during 2015–2018. The survey of tomato leaf curl New Delhi virus (ToLCNDV) was carried out in another 35 zucchini greenhouses in 2017 (not shown). (A) Almería city.

During the three consecutive campaigns of 2015–2016, 2016–2017, and 2017–2018, zucchini crops grown in 44 commercial greenhouses in the province of Almeria in SE Spain (36°45′06″ N, 2°41′04″ W) were screened for the presence of *B. tabaci* and *A. swirskii* (Figure 1). Naturally occurring predators and parasitoids of *B. tabaci* such as *Nesidiocoris tenuis* (Reuter) (Hemiptera: Miridae) and *Eretmocerus mundus* (Hymenoptera: Aphelinidae) were also evaluated. The type of whitefly management, either chemical (conventional) or integrated, was annotated for each of the 13, 22, and 23 zucchini crops that were screened during the respective crop campaigns. During the month of December 2017, 35 other zucchini greenhouses, located in the region of Níjar (East from the capital city of Almería) were monitored for the presence of ToLCNDV symptoms that typically include curling and yellowing in young leaves [21]. The data were compiled by the Andalusian Ministry of Agriculture, Livestock, Fisheries and Sustainable Development, within the Warning and Information Plant Protection Network of the Andalusia Government (Red de Alerta e Información Fitosanitaria; RAIF in Spanish) [22] and retrieved by Antonio De Pablo Gómez-Bastero (Tragsa—SEPI, Seville, Spain) on 20 March 2019.

### 2.2. General Experimental Procedures

The experiments were conducted in a greenhouse complex from May to July 2017 at the Centre IFAPA La Mojonera, Almería, Spain. The complex spanned 1500 m^2^ and consisted of one surrounding corridor and eight research compartments of approximately 100 m^2^, oriented east–west. Each compartment consisted of five rows with 15 plants each. Four hundred and fifty three-week-old seedlings (three true leaves-stage) of zucchini plants (*Cucurbita pepo* L.) cv. Victoria (HM. Clause, Spain) were transplanted into perlite grow bags with a volume of 100 liters at a density of three plants per bag. Two of these compartments (replicates) were randomly assigned to one whitefly control strategy (treatment). The three whitefly control strategies were as follows (1) “CB treatment” consisted of preinstallation of the predatory mite *A. swirskii* in zucchini seedlings; (2) “Chemical treatment” consisted of application of conventional pesticides; and (3) “IPM treatment” consisted of preinstallation of *A. swirskii* in zucchini seedlings in combination with application of pesticides compatible with the predatory mites (Table 1). Each treatment was separated from the other treatments by an empty compartment which served as a “buffer zone”. For the biological control treatment, predatory mites were supplied by Agrobio S.L. (La Mojonera, Almería, Spain) and provided in commercial paper sachets of 250 individuals supplemented with storage mites (all stages) mixed with bran. Predators were transferred to seedlings within 24 h of receipt, three days prior to transplanting, and released at the rate of ∼55 individuals per plant [20]. For the chemical treatment, two applications of conventional pesticides per week were sprayed to run-off using a pressurized sprayer. The pesticides applied under this strategy were those used commonly by growers (Table 1). For the IPM treatment, the predatory mites were applied as described above and in combination with a single application of biorational pesticides per week (Table 1). One day after transplanting, all crops were inoculated with twenty-five viruliferous whiteflies released at a height of 1.5 m from the soil into the center of each compartment to simulate a natural whitefly invasion and spread. The ToLCNDV-viruliferous whiteflies were taken from colonies maintained on zucchini plants (cv. Victoria) in insect-proof cages under controlled rearing conditions at 25 ± 3 °C and with a photoperiod of 16:8 h light/dark. The population was periodically tested for ToLCNDV to confirm the presence of virus. The biotype of the *B. tabaci* populations, was determined by partial mitochondrial cytochrome oxidase I gene sequencing as described and was found to belong to the Mediterranean cryptic species subclade Q1, following the methodology described by Gueguen et al. [23].

### 2.3. Monitoring B. tabaci and ToLCNDV Symptom Evolution in Experimental Greenhouse Compartments

Pest pressure outdoors was monitored weekly during the experiment (seven weeks) by counting captures of adult whiteflies on twelve 25 × 10 cm yellow sticky traps (two traps/compartment oriented north-south) (Agrobio S.L. La Mojonera, Almería, Spain) placed inside the greenhouse corridor at 1, 60 cm of the soil. Observations within greenhouse compartments were done every week on the next day after pesticide applications over 7 weeks. For each treatment and repetition, three zucchini leaves per plant were gently turned to reveal the leaf underside, and the numbers of *B. tabaci* adults were recorded (*n* = 450 leaves/treatment). Expression of ToLCNDV symptoms was monitored as described above during six consecutive weeks on all the zucchini plants and in every greenhouse compartment.

### 2.4. Data Analysis

The numbers of *B. tabaci* adults were expressed as insect-day accumulated values (IDA). This index proposed by Ruppel [24], was applied to evaluate the total pest impact at a given period of time. Population trends were compared by adjusting the IDA to different sigmoid functions using the Table curve 2D v 5.01 statistical software (Systat Software, Inc., San Diego, CA, USA). IDA end values were analyzed by the generalized linear model. The models were fitted by maximum likelihood estimation with the GenLin procedure with gamma errors and the log link function using the IBM SPSS version 25.0 statistical software package [25]. The significance of the model was assessed by an Omnibus test to test whether the explained variance in a data set was significantly greater than the unexplained overall variance. For the regression effect specified in the model, a Wald statistic was conducted, which is based on the linearly independent pairwise comparisons among the estimated marginal means [26]. Then, the mean values were compared pairwise at *p* = 0.05. Analysis of ToLCNDV expression was done calculating the proportions of plants that expressed symptoms of the 15 plants in each of ten rows per treatment. These proportions were used to calculate the area under disease progress curve (AUDPC) following Campbell and Madden [27]. The means and standard deviations of AUDPC values per treatment were calculated and differences between means were evaluated using the *t*-test (double sided probability, *p* < 0.05).

## 3. Results

### 3.1. Phytosanitary Balance, Pest Management, and ToLCNDV Incidence in Commercial Zucchini Crops

During the three consecutive crop seasons between 2015 and 2018, *B. tabaci* whiteflies were present in almost all monitored zucchini crops grown in commercial greenhouses form the province of Almeria. During the first and the third campaign, all of the greenhouses had this whitefly species on more than 5% of plants. Initially, none of the greenhouses had natural enemies *E. mundus* and *N. tenuis*, but a few of them had *N. tenuis* by the campaign of 2017–2018. Predatory mite *A. swirskii*, however, was found in about 69% of screened greenhouses during 2015–2016, but this ratio dropped to 36 and 35% of greenhouses during the two following years (Table 2).

During the campaign of 2015–2016, IPM was predominantly used in the monitored zucchini crops (69%), whereas the remaining greenhouses used chemical pest control. However, during the two following years, the numbers of greenhouses with chemical pest control increased to over 60% of the greenhouses, whereas less than 40% used IPM (Table 3).

**Figure 2:**
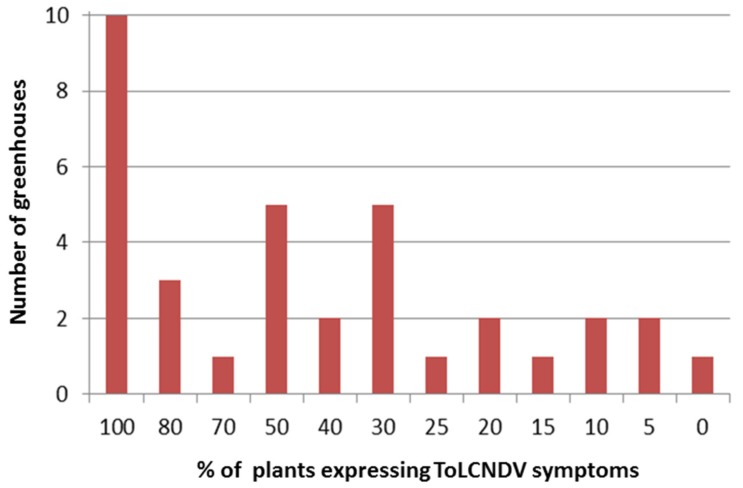
Greenhouse frequency distribution of ToLCNDV-symptomatic plants.

Visual inspection for symptoms of ToLCNDV indicated the presence of the virus in zucchini crops in 34 of 35 monitored greenhouses during the fall of 2017. More than 5% and 20% of plants had symptoms, in 34 and 28 greenhouses, respectively (Figure 2).

### 3.2. Whitefly and ToLCNDV Evolution in Experimental Greenhouse Compartments

#### 3.2.1. Whitefly

**Figure 3:**
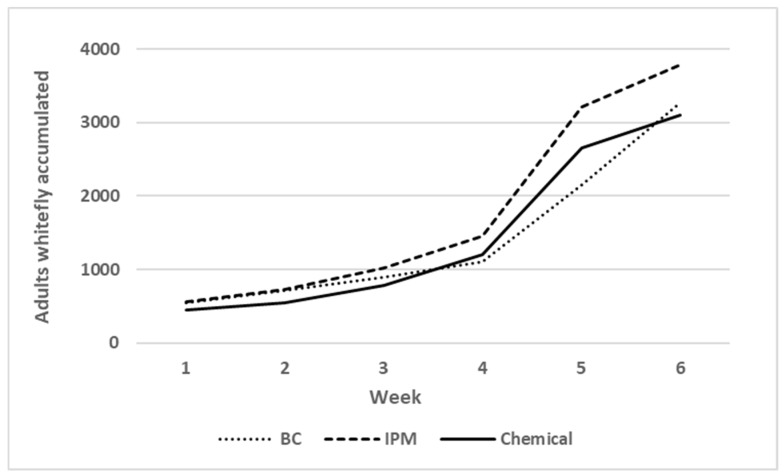
Accumulated average number of adult whitefly per yellow sticky trap outdoors of the experimental greenhouse compartments.

Temporal dynamics of whitefly population outdoors were similar in all treatments (biological, integrated, and chemical) during the seven weeks of monitoring, as indicated by number of whitefly adults accumulated on yellow sticky traps (Figure 3). Cumulative mean numbers increased intensely from week 5, rising from a mean value of about 500 whiteflies on week 1 to about 3200 on week 7 (Figure 3). However, the final number of accumulated whitefly adults in IPM treatment exceeded 3700 individuals. Thus, pest-pressure effect on zucchini plants was very similar on biological and chemical compartments, but was higher on IPM compartments.

With respect to the dynamics of whitefly populations indoors, the factor (treatment) of the model had a significant effect on the value of IDA at the end of the trial (Omnibus test, likelihood ratio *χ*^2^ = 52.454, *d.f.* = 2; *p* < 0.001) (Table 4). The biological control strategy showed the lowest whitefly population at the end of the crop, followed by the integrated and the chemical strategy (*p* < 0.001) (Table 4). The lowest pest population (not exceeding 12.27 whiteflies per leaf) was observed under the biological treatment in week 7, followed by that under the integrated treatment with values not exceeding 24.46 whiteflies per leaf. Conversely, higher numbers of IDA of *B. tabaci* were observed under the chemical strategy, which induced the higher pest population at the end of the crop, with 41.97 whiteflies per leaf in week 7.

**Figure 4:**
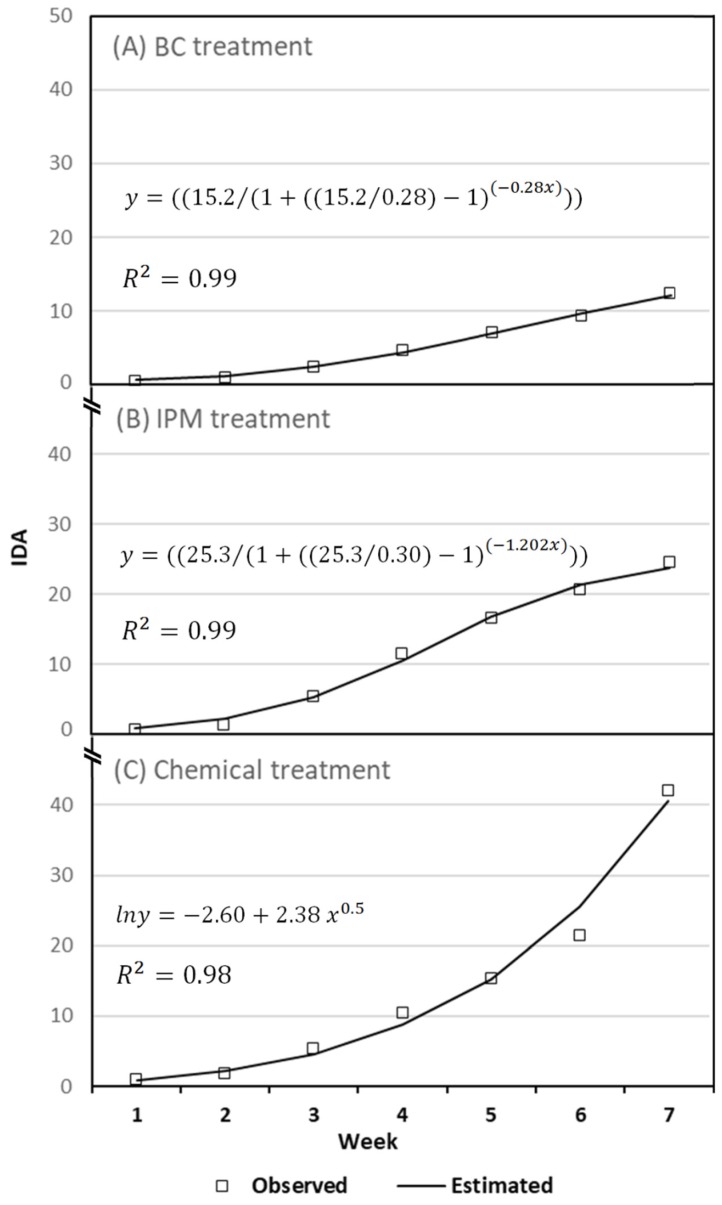
Mean insect-day accumulated values (IDA) of adults of *B. tabaci* under biological (**A**), integrated (**B**), and chemical (**C**) control strategies from experimental data (□) and predicted values (▬) by nonlinear functions. The cumulative experimental data under biological (**A**) and integrated (**B**) strategies fitted to a logistic equation and that under chemical strategy (**C**), to an exponential equation.

The accumulated numbers of *B. tabaci* adults under the biological (F = 165; *d.f.* = 2; *p* < 0.0001; R^2^ = 0.99) and integrated (F = 898.3, *d.f.* = 2, *p* < 0.0005; R^2^ = 0.99) strategy fitted a logistic function fairly well. Quantitative data analysis of *B. tabaci* indicated that both biological and integrated treatments were important in reducing and controlling the virus vector at the end of the crop. By contrast, the evolution of *B. tabaci* accumulated numbers under chemical treatment was well described by an exponential function (F = 653.9, *d.f.* = 1, *p* < 0.0000; R^2^ = 0.88) (Figure 4). In this case, estimated numbers of adults of *B. tabaci* constantly increased along the time, particularly since week 3, indicating that whitefly was not being controlled successfully by exclusively using a chemical control.

#### 3.2.2. ToLCNDV Symptom Evolution

**Figure 5:**
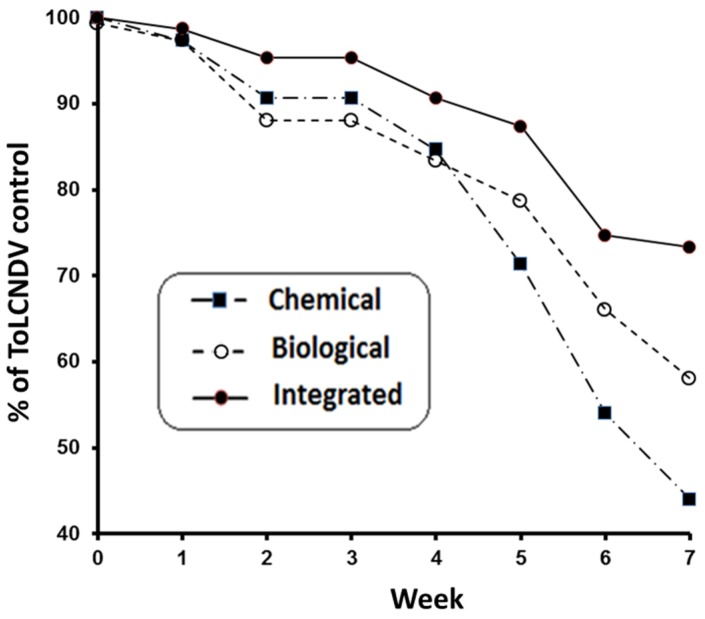
Control of ToLCNDV in zucchini under chemical, biological, and integrated management.

Between weeks 1 and 2 and following the release of viruliferous whiteflies, increasing numbers of zucchini plants expressed the typical symptoms of ToLCNDV. Already during the first few weeks of the trial, zucchini crops under the integrated strategy showed fewer plants expressing symptoms when compared with plants under biological or chemical control. Plants under the later strategy expressed more symptoms when compared with biological control after four weeks of infection (Figure 5). The maximum proportions of symptomatic plants under the different control strategies were 0.56, 0.42, and 0.27, under chemical, biological, and integrated whitefly management, respectively. The means and standard deviations of calculated AUDPC values were 10.1 ± 4.8, 8.4 ± 4.6, and 5.04 ± 4.7 for ToLCNDV under, respectively, chemical, biological, and integrated whitefly management. AUDPC means of symptomatic plants under chemical and integrated strategies were significantly different (Table 5).

## 4. Discussion

Together with two-spotted spider mite (*Tetranychus urticae* Koch) (Acarina: Tetranychidae) and western flower thrips (*Frankliniella occidentalis* Pergande) (Thysanoptera, Thripidae), the whitefly *B. tabaci* constitutes the main pest of zucchini in the Mediterranean region [28]. Consequently, we found *B. tabaci* commonly infesting this crop species in greenhouses from SE Spain during the three consecutive years of study (Table 2). No silverleaf symptoms were observed, consistent with previous results that characterized the whitefly as cryptic species MedQ1 [5].

Of the complex of beneficial insects that are used for *B. tabaci* control in protected horticultural crops in SE Spain, the parasitoid *E. mundus* and the predator *N. tenuis* were found only in a few greenhouses during the period of monitoring (Table 2). Instead, *A. swirskii* was frequently observed especially during the first and the last year of monitoring. This natural enemy has been commercially available in Spain since 2007, and is increasingly used to control pests in pepper and recently also in cucumber crops [18]. However, biological control-based IPM in zucchini is applied on a very limited scale, i.e., only 1050 ha, which is 13.2% of the greenhouse area dedicated to zucchini. This is in contrast with solanaceous crops in Almería, where natural enemies are intensively applied in pepper (100% of crops) and tomato (80%) (crop season of 2016/2017) (Junta de Andalucía, Consejeria Agricultura y Pesca). Our results seem to confirm this trend: farmers did not rely on IPM for whitefly control in zucchini, and chemical treatments were progressively preferred during the campaigns between 2015 and 2018 (Table 3). Besides the emerging threat of ToLCNDV in zucchini, another reason explaining the limited application of biological control is that predatory mites lack the time for populations to increase on this short-cycle crop, and thus may fail to provide good biological control of pests. In this sense, the preventative installation of *A. swirskii* in young plants during the nursery period could help to overcome the problem of mite establishment in zucchini crops. This technique has proven to be very successful in semifield conditions [20].

Experimental data from the three treatments evaluated for whitefly control showed that those that involved the use of the predatory mite, i.e., biological and IPM treatments, resulted in better pest control (Table 4, Figure 4). The whitefly population growth fitted a logistic function fairly well in both cases. *A. swirskii* is an efficient predator of eggs and crawlers of *B. tabaci* [14,29] and can suppress populations of the tobacco whitefly [30]. Previous results have shown that *B. tabaci* adults avoid plants with preinstalled *A. swirskii* (colonization) and therefore contributed to further control of *B. tabaci* populations (reproduction) [20]. Although both biological and IPM strategies were ultimately effective in suppressing the whiteflies, biological treatment resulted in a significantly better pest control when compared with IPM. This might be due to the higher pest pressure onto the IPM compartments during the experiment (Figure 3). On the other hand, the increase of the whitefly population on plants with the chemical treatment was described by an exponential growth equation, and no further decrease in the pest population could be observed during the experimental period (Figure 4). Our results are consistent with those from other studies where *B. tabaci* has been difficult to control with conventional insecticides in horticultural production systems [8,31]. In fact, *B. tabaci* is able to develop resistance to synthetic pesticides [32]. Moreover, the pesticide switching method used in this context (conventional management) will result in multiple pesticide resistance, and lead to negative effects on whitefly control. In the past, chemical pest control programs applied in horticulture greenhouses from Almería have proved totally unsustainable [33].

*B. tabaci* can transmit 12 viruses in countries of the West-Mediterranean, all of which have been described in Spain [34]. One of the recently introduced viruses is ToLCNDV, which infects solanaceous and cucurbitaceous crops, but is especially aggressive in zucchini. We found symptoms of ToLCNDV in all but one of the 35 monitored greenhouses. Since its introduction in 2013, this virus is the main disease in the region. Juarez et al. [11] detected ToLNDV in 182 of 191 collected zucchini samples in the neighboring Autonomous Community of Murcia during five consecutive cropping seasons (2012–2016). ToLCNDV symptoms are dependent on the cultivar and growing conditions, although common symptoms may include curling and yellowing in young leaves of zucchini plants, and vein swelling in cucumber plants [35].

The experimental comparison of the three different whitefly control strategies, showed that the chemical strategy was the least efficient in controlling the evolution of ToLCNDV in zucchini (Table 5, Figure 5). It has also been postulated that synthetic chemicals may affect the behavior of vectors, and hence of virus transmission [36]. Virus acquisition and transmission by insects often occurs too fast for insecticides to control viral dispersal. Pesticide treatment has been shown to be inefficient in controlling tospoviruses transmitted by thrips [37], and even enhances the dispersal of potyviruses transmitted by aphids [38]. This should be further studied in the case of whiteflies and the viruses they transmit.

Insect predators and parasitoids can be effective in controlling pests, and consequently putative vectors of viruses, in greenhouse crops [39]. However, they are considered unable to control transmission of viruses [40]. In the case of ToLCNDV in zucchini, *A. swirskii* does not prevent primary infections from invading viruliferous whiteflies, but this predator controls significantly secondary spread of the virus from infested and infected host plants [20]. The experimental comparison of control strategies in the present paper showed that the use of integrated whitefly management led to the best control of ToLCNDV dispersal (Figure 5, Table 5). Likely, pesticides used under the IPM strategy reduced the primary infection of zucchini plants without compromising the biological control of the secondary virus spread. Results suggest that insecticides were harmful to invading whitefly adults and therefore reduced the overall primary virus infection. Moreover, these insecticides that were effective against *B. tabaci* adults were relatively compatible with *A. swirskii*, which preys on eggs and crawlers of *B. tabaci*, resulting in a strong decrease of new viruliferous whiteflies and, consequently, a significant reduction of virus incidence. However, this study does not prove that *A. swirskii* can reduce virus incidence on a large commercial scale and under true greenhouse conditions. Future experiments should confirm this hypothesis. Note that only the simplest *B. tabaci*–*A. swirskii* system has been employed in the present work to address the key issue related to whitefly control and virus suppression. Under commercial greenhouse conditions, several variables may affect functional responses of the *A. swirskii* which have not been included in this experimental work, and which could significantly affect the outcome of whitefly control and virus spread. Some of these factors are a varying environment (temperature and humidity), presence of alternative prey, intraguild predation, cultural practices, and plant management practices (e.g., irrigation, fertilization, and disease control). All these variables cannot be completely evaluated under noncommercial conditions, even in experimental greenhouses. Thus, the results emphasize the need to adopt a biological control-based IPM which will reduce ToLCNDV incidence in commercial zucchini crops, whilst reducing the harms that accompany the use of chemicals, including those associated with environmental pollution, and the widespread resistance and resurgence of *B. tabaci*.

## 5. Conclusions

The use of chemical pesticides is the main strategy adopted by growers in commercial zucchini greenhouses from SE Spain to control *Bemisia tabaci* whiteflies; however ToLCNDV is widespread. Under experimental conditions, a biological control strategy involving the predatory mite *Amblyseius swirskii* in source plants decreased the whitefly abundance in zucchini crops. Biological control-based IPM was the best strategy for virus suppression. The exclusion of invading whitefly adults by using IPM-compatible pesticides reduced the primary spread of the whitefly-transmitted virus, whereas the predation of eggs and crawlers (first nymphal instar) by the mites reduced secondary virus spread. Finally, the maximum values of whitefly adults as well as of virus symptoms were found in crops under the conventional (chemical), strategy, suggesting that whitefly control by pesticides was the worst strategy for pest control and virus suppression. Therefore, the biological control-based IPM strategy proposed here might reduce the abuse of pesticides and efficiently address the virus risk that limits current commercial zucchini production in SE Spain.

## Figures and Tables

**Table 1:** Biological, chemical, and integrated treatment schedule applied in the experimental greenhouse compartments.

Dates	Biological	Chemical ^1^	Integrated ^1^
3 days before transplanting	*Amblyseius swirskii* (55 individuals/plant)		*Amblyseius swirskii* (55 individuals/plant)
Weeks 1, 5		acetamiprid + phosphoric soap	pymetrozine
Weeks 2, 6		pymetrozine + phosphoric soap	phosphoric soap
Weeks 3, 7		acetamiprid + phosphoric soap	phosphoric soap
Week 4		spirotetramat + azadirachtin	spirotetramat

^1^ pesticides were applied against whitefly at the recommended doses.

**Table 2:** Number (% of total) of zucchini crops with whitefly (*Bemisia tabaci*), commercial release of *Amblyseius swirskii* and naturally occurring parasitoid and predator (*Eretmocerus mundus* and *Nesidiocoris tenuis*) on more than 5% of plants.

Campaign	Greenhouses (*n*)	*B. tabaci*	*A. swirskii*	*E. mundus*	*N. tenuis*
2015–2016	13	12 (92.3%)	9 (69.2%)	0 (0.0%)	0 (0.0%)
2016–2017	22	19 (86.4%)	8 (36.4%)	0 (0.0%)	3 (13.6%)
2017–2018	23	22 (95.6%)	8 (34.8%)	0 (0.0%)	2 (8.7%)

**Table 3:** Number (% of total) of zucchini crops with chemical or integrated pest control of *B. tabaci*.

Campaign	Greenhouses (*n*)	Pest Management (*n* (%))	
		Chemical	Integrated
2015–2016	13	4 (30.8%)	9 (69.2%)
2016–2017	22	14 (63.6%)	8 (36.4%)
2017–2018	23	14 (60.9%)	9 (39.1%)

**Table 4:** IDA (insect-day accumulated) values for *B. tabaci* at the end of the zucchini cultivation (week 7) under three different whitefly control strategies. Means within a column followed by the same letter are not significantly different a *p* = 0.05; Wald Chi-Squared Test = 43.21, gl = 2, *p* < 0.0001.

Strategy	Mean	SE	95% Confidence Interval (Wald)
Biological	12.27a	1.43	9.75–15.44
Integrated	24.46b	2.86	19.45–30.78
Chemical	41.97c	4.92	33.36–52.80

**Table 5:** Mean values of area under the disease progress curve (AUDPC) of ToLCNDV-symptomatic plants in experimental zucchini greenhouse compartments.

Difference AUDPC Means	M1–M2	95% Confidence Interval (Wald)	*t*-Difference	*p* Value
Biological vs. Integrated	3.36	1.02–7.77	1.607	>0.0500
Chemical vs. Integrated	5.06	0.57–9.54	2.369	0.0292
Chemical vs. Biological	2.06	−2.34–6.41	0.980	>0.0500
